# Email as an Encumbrance to Physician-patient Communication

**DOI:** 10.7759/cureus.3816

**Published:** 2019-01-03

**Authors:** William C Welch, Melissa S Mathew, Rachel L Welch, Brendan J McShane

**Affiliations:** 1 Neurosurgery, University of Pennsylvania / Pennsylvania Hospital, Philadelphia, USA

**Keywords:** email, doctor patient communication, doctor patient relationship

## Abstract

Physician-patient interaction through email poses several concerns regarding the security, efficiency, and misinterpretation of critical information. Incoming emails received by a single university-based physician in 2013 were analyzed in order to determine whether a general non-patient specific email is appropriate for patient use. Emails received were divided into seven categories: Informational, Academic, Advertisement, Organization/Department/ University, Mission Critical, Personal, and Patient. A total of 9,102 emails were received and read by the physician, with an average of 25 emails per day, out of which 823 (9%) emails were directly sent by patients. The total time spent reading emails was five days, seven hours, and 24 minutes. General email is not an effective means of streamlining physician-patient communication. Non-essential emails, which represent a majority of incoming messages, decrease the productivity of physicians and prevent them from responding to urgent messages in a timely manner. Additionally, this creates the chance for critical patient information getting lost with the volume of received emails. This could be detrimental to patient care and satisfaction. Recently, an online portal was instated to provide a method of secure communication, and less than five patient emails were received in the physician’s personal email since then.

## Introduction

Follow-up appointments tend to be scheduled based on calendar availability rather than on patient need, leading to an increased necessity for doctor-patient communication between appointments [1]. Patients frequently seek to use email to communicate with their healthcare providers [2]. Physicians’ are primarily concerned about: 1) the lack of security measures in sending/receiving emails, 2) increased time commitment and effort required by doctors to read and respond to emails, 3) risk and liability stemming from a miscommunication or misunderstanding of vital information, 4) likelihood of patients consulting via email for trivial issues, and 5) increased possibility of crucial information being lost within the volume of emails that flood a physician’s inbox [2-3].

When personal email is used, there are insufficient defenses to address security concerns [2]. The Federal Health Insurance and Portability and Accountability Act (HIPAA) requires that electronic communication regarding patient health be protected at all times [4]. This means that electronic messages need to be encrypted or delivered through a secure portal. Personal email accounts do not offer these security measures [5]. The potential for sensitive patient information being delivered through non-secure emails puts the physician at an increased risk of litigation or violation of HIPAA [2]. Out of all the patients that use email to contact their physicians, it is estimated that up to 90% tend to send critical information [4]. Despite these security issues, approximately 1%-10% of patients in the United States communicate with their physicians through email [6].

The demanding workload of physicians can be further hindered by unnecessary emails. Hospitalists reported that feeling overworked often prevents them from being able to fully discuss treatment options with their patients, and over 20% believe that stress from their average workload could have led to patient transfers, morbidity, or mortality [7]. Workload management is key for the optimal performance and safety of doctors and patients. Interruptions like having to stop to read and respond to emails contribute to an increased workload [8]. If physicians wait until after seeing patients to tackle their inbox, the volume of emails can become overwhelming, and patient satisfaction decreases as response times increases [6].

When a patient does reach out to his or her physician via email, it usually relays vital and medically relevant information that may require a response [9]. It is crucial that these messages are addressed in a timely fashion and not lost amidst other messages. Due to the growing uses of email, most messages swarming a person’s inbox are unnecessary. This causes the individual on the receiving end to spend more time sifting through emails in search of significant and critical emails. Additionally, some emails may have important messages inside, which are hidden behind “courtesy.” These factors make emailing an inefficient means of communicating with a health care provider.

Physicians already spend a lot of time reading and responding to professional emails. This reduces available time for other endeavors. Our goal here is to quantify the time a physician spends reading emails and to compare the volume of patient emails to the full range of emails crowding the inbox. We thus hope to determine whether a general, non-patient-specific email inbox is appropriate for patient use.

## Materials and methods

We analyzed the number of emails delivered to an account accessible to both patients and university members. The data are the emails received by a single university-based physician received during the 2013 calendar year. Emails were divided into seven categories: Informational, Academic, Advertisement, Organization/Department/University, Mission Critical, Personal, and Patient. Informational emails provided general organizational information. Advertisement emails were sent by companies to promote their products or inform about ongoing sales and promotions. Organization/department/university emails were intended to notify staff about upcoming events or activities. Mission-critical emails were messages requiring immediate attention regarding organizational occurrence or a patient’s condition but were not sent directly by patients. Personal emails addressed the physician’s life outside of the hospital. Patient emails came directly from the patient, containing information about the patient’s conditions or questions about care. The total time spent on opening and reading emails was recorded in minutes. The total time spent on responding to emails was not included.

## Results

In 2013, a total of 9,102 emails were received by the physician, with an average of 25 emails per day. A total of 823 (9%) emails were directly sent by patients. The total time spent reading emails was five days, seven hours, and 24 minutes. The mean weekly reading time was two hours and 27 minutes.

**Figure 1:**
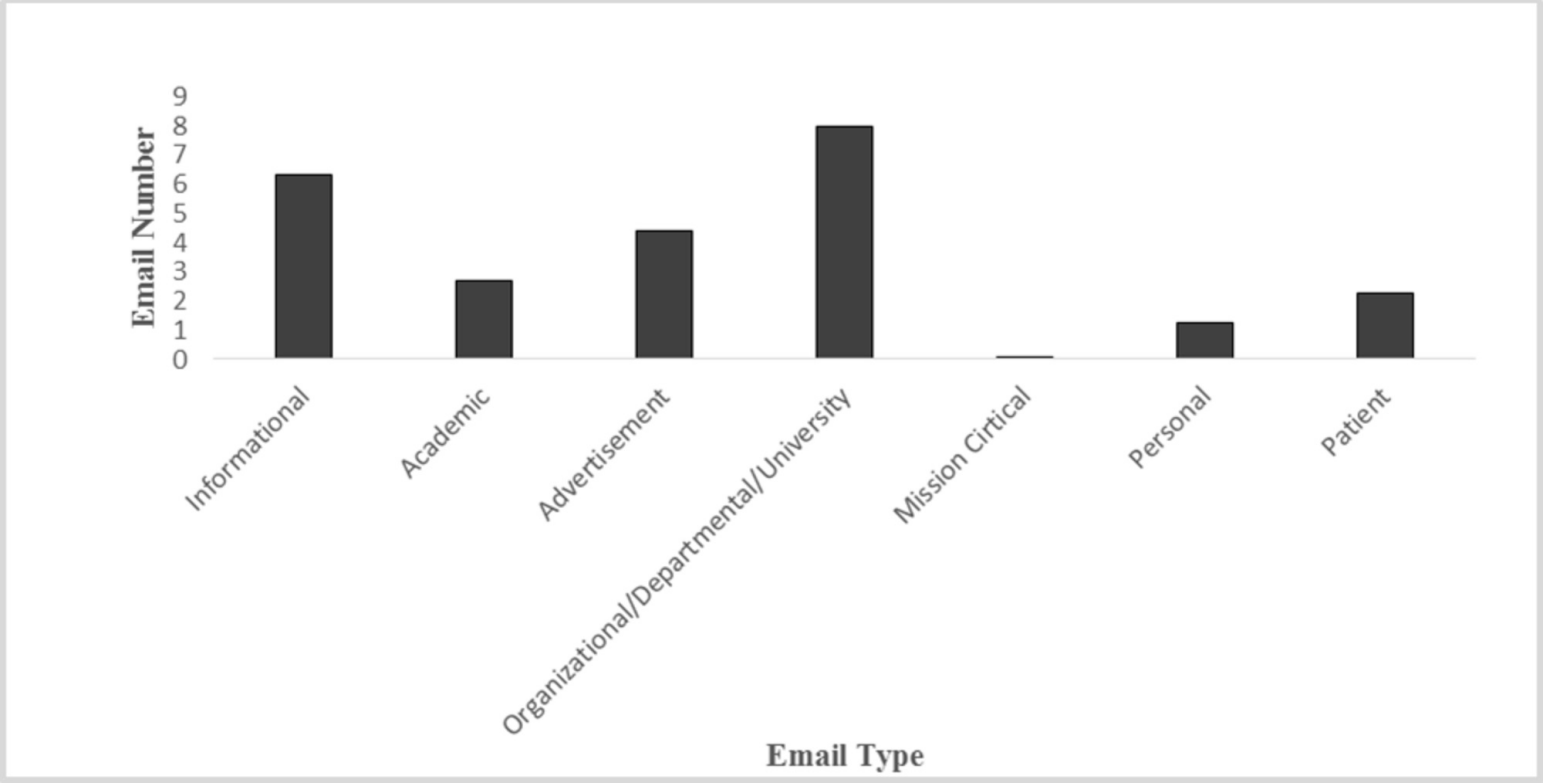
Average Number of Emails Received per Day per Email Type Average number of emails received daily, sorted by email type

**Figure 2:**
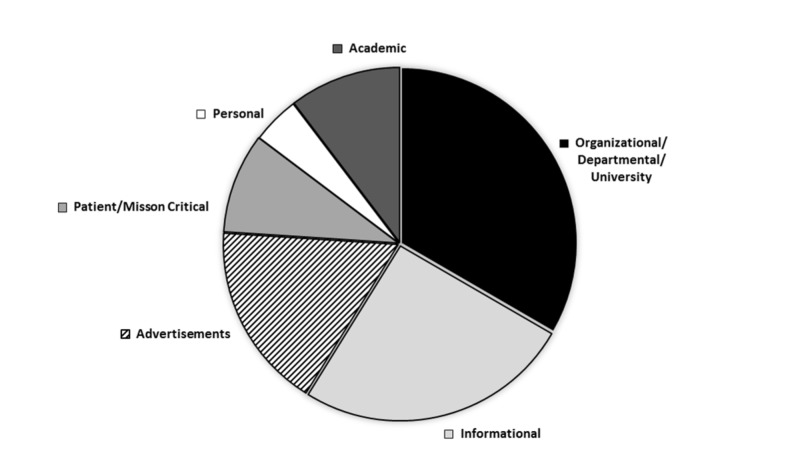
Percentages of Email Types Percentage of total emails received for each of the seven categories: Informational, Academic, Advertisement, Organization/Department/University, Mission Critical, Personal, and Patient

Organizational/departmental/university emails account for 29.32% of the total received emails, averaging eight emails per day. Informational, advertisement, academic, and personal emails represent 22.41% (6.3 per day), 15.10% (2.7 per day), 9.09% (4.4 per day), and 3.85% (1.3 per day), respectively. Patient emails and mission-critical emails were grouped together because both categories contain vital information relating to patient care. Collectively, these categories represented 8.13% of the total emails (Figures 1-2).

## Discussion

Physicians spend a great deal of time on opening and reading an increasing number of emails, which decreases physician productivity. An analysis of the physician’s inbox shows that the bulk of the received emails were organizational/departmental/ university, informational, and advertisement emails. Patient/mission-critical emails represented a very small percentage (Figure 2). Reading through all these emails took an average of two hours and 23 minutes per week. A majority of these emails do not affect patient care nor are they mission critical. In addition, this exorbitant amount of time physicians dedicate to email does not include the time taken to reply to each email. Since the time spent on reading emails affects either the professional life or the personal life of doctors, measures should be taken to decrease the amount of non-essential emails. This will also help doctors communicate with their patients online more often.

We believe that general email is not an effective means of streamlining physician-patient communication. Non-essential emails, which represent a majority of incoming messages, decrease the productivity of physicians and prevent them from responding to urgent messages in a timely manner. Additionally, this invites the chance for critical patient information to get lost with the volume of received emails, which is potentially detrimental to patient care and satisfaction.

Our institution recently instated a portal that allows patients to send non-urgent messages to their physicians to facilitate constructive conversation. These messages are linked to the patient’s electronic medical records and notify the doctor and their team when a message is received. This portal allows both the physician and nurse practitioner to respond quickly and efficiently to the patient's concern. Since the instillation of this portal, less than five patient emails have been received by the physician in their personal inbox per year. Encouraging patients to utilize such portals and taking steps to reduce the number of unnecessary emails in the physician’s general email inbox can drastically improve patient-physician communication.

Solutions

The following are some quick and easy techniques that physicians can use to decrease the number of unwanted incoming messages and use their reading time more efficiently: 1) avoid using a professional email account when making online purchases or signing up for promotions, 2) request that senders indicate the significance of the email in the subject line by marking if it is a real emergency, and 3) avoid hitting “reply all” when responding to messages unless everyone in the email truly requires that piece of information. Eliminating promotional emails from product marketers to a professional email account will significantly decrease the number of emails received and, as a result, decrease the reading time as well. Assigning importance in the subject line of the email will help doctors skim their emails in search of critical information and help those in need as soon as possible.

Limitations

The data was only collected from one individual, and it may not be representative of the experience of all physicians. Additionally, the fact that this physician practices in a university-affiliated hospital means that the additional university-related emails that he receives would not be received by physicians who are not working in a teaching hospital.

Future research

The time physicians spend responding to emails was not quantified in this study but can be explored in the future to get a better idea of time spent on emails. Additionally, a statistical analysis of the contents of the inboxes of multiple physicians can reveal whether the number and types of incoming emails vary significantly.

## Conclusions

Physicians spend an extensive amount of time on reading emails and even more time when they must respond to them, but a majority of the emails they receive are not directly related to their patient care. If the influx of unwanted emails is minimized, essential emails are flagged important in the subject line, and patients are encouraged to use secure portals, there is potential to better utilize email as a means of fostering more communication between doctors and their patients.
